# Supplementary material for: Rapid and specific degradation of endogenous proteins in mouse models using auxin-inducible degrons
Source: eLife. 2022 Jun 23;11:e77987. doi: 10.7554/eLife.77987 (PMC9273210; doi:10.7554/eLife.77987)
Supplement: Supplementary file 3. [file elife-77987-supp3.docx]

**Appendix S1: Oligonucleotide details**

**Single Stranded Repair templates used for Easi-CRISPR. Exogenous sequences (linker:AID:Clover) are highlighted in green.**

***NCAPH* gRNA target sequence:** CCGCTGCAGACGTCTCAAAG

***NCAPH*-AID-mClover Repair Template ssODN**

TGTTCTCAAGTCTATCAAAGCCTGTGTTTCTTTCTTCTCTTCCCTCAGAATCTGAAGCTAGAAGGCACAGAGGATCTTTCTGATGTTCTGGTGATGCAAGGGGACGGGGGAGCAGGAGGCGTCGACGGTATCGATCCTAAAGATCCAGCCAAACCTCCGGCCAAGGCACAAGTTGTGGGATGGCCACCGGTGAGATCATACCGGAAGAACGTGATGGTTTCCTGCCAAAAATCAAGCGGTGGCCCGGAGGCGGCGGCGTTCGTGAAGAAGCTTGATGTGAGCAAGGGCGAGGAGCTGTTCACCGGGGTGGTGCCCATCCTGGTCGAGCTGGACGGCGACGTAAACGGCCACAAGTTCAGCGTCCGCGGCGAGGGCGAGGGCGATGCCACCAACGGCAAGCTGACCCTGAAGTTCATCTGCACCACCGGCAAGCTGCCCGTGCCCTGGCCCACCCTCGTGACCACCTTCGGCTACGGCGTGGCCTGCTTCAGCCGCTACCCCGACCACATGAAGCAGCACGACTTCTTCAAGTCCGCCATGCCCGAAGGCTACGTCCAGGAGCGCACCATCTCTTTCAAGGACGACGGTACCTACAAGACCCGCGCCGAGGTGAAGTTCGAGGGCGACACCCTGGTGAACCGCATCGAGCTGAAGGGCATCGACTTCAAGGAGGACGGCAACATCCTGGGGCACAAGCTGGAGTACAACTTCAACAGCCACAACGTCTATATCACGGCCGACAAGCAGAAGAACGGCATCAAGGCTAACTTCAAGATCCGCCACAACGTTGAGGACGGCAGCGTGCAGCTCGCCGACCACTACCAGCAGAACACCCCCATCGGCGACGGCCCCGTGCTGCTGCCCGACAACCACTACCTGAGCCATCAGTCCGCCCTGAGCAAAGACCCCAACGAGAAGCGCGATCACATGGTCCTGCTGGAGTTCGTGACCGCCGCCGGGATTACACATGGCATGGACGAGCTGTACAAGTGAGTACACTTTGAGACGTCTGCAGCGGGATGCCCATGGCTTCCTGGTGGCCGTGGAGAGGTGCTGGGCCTGTAGCGTGGCTATAGCCAACCACCACGGTGCTGG

***NCAPH2* gRNA target sequence:** ATCCATGGCCCAGCCTTGAG

***NCAPH2*-AID-mClover Repair Template ssODN**

CCAGGACTGGAGGCAGCTGTGGACACAATGTCTCTGAGACTGCTCACACACCAGCGAGCCCACACCCGCTTCCAGACCTATGCTGCACCATCCATGGCCCAGCCTGGGGGAGCAGGAGGCGTCGACGGTATCGATCCTAAAGATCCAGCCAAACCTCCGGCCAAGGCACAAGTTGTGGGATGGCCACCGGTGAGATCATACCGGAAGAACGTGATGGTTTCCTGCCAAAAATCAAGCGGTGGCCCGGAGGCGGCGGCGTTCGTGAAGAAGCTTGATGTGAGCAAGGGCGAGGAGCTGTTCACCGGGGTGGTGCCCATCCTGGTCGAGCTGGACGGCGACGTAAACGGCCACAAGTTCAGCGTCCGCGGCGAGGGCGAGGGCGATGCCACCAACGGCAAGCTGACCCTGAAGTTCATCTGCACCACCGGCAAGCTGCCCGTGCCCTGGCCCACCCTCGTGACCACCTTCGGCTACGGCGTGGCCTGCTTCAGCCGCTACCCCGACCACATGAAGCAGCACGACTTCTTCAAGTCCGCCATGCCCGAAGGCTACGTCCAGGAGCGCACCATCTCTTTCAAGGACGACGGTACCTACAAGACCCGCGCCGAGGTGAAGTTCGAGGGCGACACCCTGGTGAACCGCATCGAGCTGAAGGGCATCGACTTCAAGGAGGACGGCAACATCCTGGGGCACAAGCTGGAGTACAACTTCAACAGCCACAACGTCTATATCACGGCCGACAAGCAGAAGAACGGCATCAAGGCTAACTTCAAGATCCGCCACAACGTTGAGGACGGCAGCGTGCAGCTCGCCGACCACTACCAGCAGAACACCCCCATCGGCGACGGCCCCGTGCTGCTGCCCGACAACCACTACCTGAGCCATCAGTCCGCCCTGAGCAAAGACCCCAACGAGAAGCGCGATCACATGGTCCTGCTGGAGTTCGTGACCGCCGCCGGGATTACACATGGCATGGACGAGCTGTACAAGTAAGTGGACAGCACTGAGGCAGGGGTGGAAAGTAGTATATACCTGGAGGTCTTTGCCCCTAATGTGCTATGGGGCCATTCACTCCAGTGCTGCCTCCTGGCTGGCCTA

*Rosa26* sgRNA target sequence: GACTCCAGTCTTTCTAGAAGA

Sequence details for the donor template used to generate the LSL-Tir1-9myc insertion at Rosa26.

LOCUS Exported 13430 bp DNA linear UNA 31-JUL-2019

DEFINITION .

ACCESSION .

VERSION .

KEYWORDS .

SOURCE natural DNA sequence

ORGANISM unspecified

REFERENCE 1 (bases 1 to 13430)

AUTHORS .

TITLE Direct Submission

JOURNAL Exported Jan 10, 2022 from SnapGene Viewer 5.3.2

https://www.snapgene.com

FEATURES Location/Qualifiers

source 1..13430

/organism="unspecified"

/mol_type="genomic DNA"

misc_feature 271..1106

/locus_tag="R26 5arm"

/label=R26 5arm

/note="color: #00ffff"

misc_feature 1096..1106

/locus_tag="5arm r"

/label=5arm r

/note="color: #00ffff"

misc_feature 2019..3646

/locus_tag="CAG Promoter"

/label=CAG Promoter

/note="color: #ff001e"

misc_feature 2019..2306

/locus_tag="CAG Enhancer"

/label=CAG Enhancer

/note="color: #00ffff"

misc_feature 3652..3685

/locus_tag="LoxP"

/label=LoxP

/note="color: #00ffff"

misc_feature 3686..3706

/locus_tag="SeqF"

/label=SeqF

/note="color: #fffd0f"

misc_feature 3686..3693

/locus_tag="Loxp"

/label=Loxp

/note="color: #00ffff"

misc_feature 3759..3766

/locus_tag="AsiSI"

/label=AsiSI

/note="color: #00ffff"

misc_feature 3767..3772

/locus_tag="MluI"

/label=MluI

/note="color: #68ff25"

misc_feature 3773..5629

/locus_tag="osTIR1"

/label=osTIR1

/note="color: #00ffff"

misc_feature 5645..5650

/locus_tag="MluI(1)"

/label=MluI

/label=MluI(1)

/note="color: #68ff25"

misc_feature 6200..7010

/locus_tag="R26 3arm"

/label=R26 3arm

/note="color: #00ffff"

misc_feature 7011..7047

/locus_tag="3arm"

/label=3arm

/note="color: #00ffff"

ORIGIN

1 caccgcatta ccctgttatc cctagcggca ggccctccga gcgtggtgga gccgttctgt

61 gagacagccg ggtacgagtc gtgacgctgg aaggggcaag cgggtggtgg gcaggaatgc

121 ggtccgccct gcagcaaccg gagggggagg gagaagggag cggaaaagtc tccaccggac

181 gcggccatgg ctcggggggg ggggggcagc ggaggagcgc ttccggccga cgtctcgtcg

241 ctgattggct tcttttcctc ccgccgtgtg tgaaaacaca aatggcgtgt tttggttggc

301 gtaaggcgcc tgtcagttaa cggcagccgg agtgcgcagc cgccggcagc ctcgctctgc

361 ccactgggtg gggcgggagg taggtggggt gaggcgagct ggacgtgcgg gcgcggtcgg

421 cctctggcgg ggcgggggag gggagggagg gtcagcgaaa gtagctcgcg cgcgagcggc

481 cgcccaccct ccccttcctc tgggggagtc gttttacccg ccgccggccg ggcctcgtcg

541 tctgattggc tctcggggcc cagaaaactg gcccttgcca ttggctcgtg ttcgtgcaag

601 ttgagtccat ccgccggcca gcgggggcgg cgaggaggcg ctcccaggtt ccggccctcc

661 cctcggcccc gcgccgcaga gtctggccgc gcgcccctgc gcaacgtggc aggaagcgcg

721 cgctgggggc ggggacgggc agtagggctg agcggctgcg gggcgggtgc aagcacgttt

781 ccgacttgag ttgcctcaag aggggcgtgc tgagccagac ctccatcgcg cactccgggg

841 agtggaggga aggagcgagg gctcagttgg gctgttttgg aggcaggaag cacttgctct

901 cccaaagtcg ctctgagttg ttatcagtaa gggagctgca gtggagtagg cggggagaag

961 gccgcaccct tctccggagg ggggagggga gtgttgcaat acctttctgg gagttctctg

1021 ctgcctcctg gcttctgagg accgccctgg gcctgggaga atcccttccc cctcttccct

1081 cgtgatctgc aactccagtc tttctaggcg cgcccgggct gcagatctgt agggcgcagt

1141 agtccagggt ttccttgatg atgtcatact tatcctgtcc cttttttttc cacagctcgc

1201 ggttgaggac aaactcttcg cggtctttcc agtggggatc gacggtatcg ataagctggc

1261 cgctctagtg gccgtacggg cccacctgcc gggccactta attaaattta aatcacaatt

1321 ccagctgagc gccggtcgct accattacca gttggtctgg tgtcaaaaat aataataacc

1381 gggcaggggg gatctgcatg gatctttgtg aaggaacctt acttctgtgg tgtgacataa

1441 ttggacaaac actccgaggc ggatcacaag catacctaca gagatttaaa gctctaaggt

1501 aaatataaaa tttttaagtg tataatgtgt taaactactg attctaattg tttgtgtatt

1561 ttagattcca acctatggaa ctgatgaatg ggagcagtgg tggaatgcca gatccagaca

1621 tgataagata cattgatgag tttggacaaa ccacaactag aatgcagtga aaaaaatgct

1681 ttatttgtga aatttgtgat gctattgctt tatttgtaac cattataagc tgcaataaac

1741 aagttaacaa caacaattgc attcatttta tgtttcaggt tcagggggag gtgtgggagg

1801 ttttttaaag caagtaaaac ctctacaaat gtggtatggc tgattatgat ctgcggccgt

1861 gctagcgctt aagcttgaag ttcctattcc gaagttccta ttctctagaa agtataggaa

1921 cttcggcgcg ccggatctga cattgattat tgactagtta ttaatagtaa tcaattacgg

1981 ggtcattagt tcatagccca tatatggagt tccgcgttac ataacttacg gtaaatggcc

2041 cgcctggctg accgcccaac gacccccgcc cattgacgtc aataatgacg tatgttccca

2101 tagtaacgcc aatagggact ttccattgac gtcaatgggt ggagtattta cggtaaactg

2161 cccacttggc agtacatcaa gtgtatcata tgccaagtac gccccctatt gacgtcaatg

2221 acggtaaatg gcccgcctgg cattatgccc agtacatgac cttatgggac tttcctactt

2281 ggcagtacat ctacgtatta gtcatcgcta ttaccatggg tcgaggtgag ccccacgttc

2341 tgcttcactc tccccatctc ccccccctcc ccacccccaa ttttgtattt atttattttt

2401 taattatttt gtgcagcgat gggggcgggg gggggggggg cgcgcgccag gcggggcggg

2461 gcggggcgag gggcggggcg gggcgaggcg gagaggtgcg gcggcagcca atcagagcgg

2521 cgcgctccga aagtttcctt ttatggcgag gcggcggcgg cggcggccct ataaaaagcg

2581 aagcgcgcgg cgggcgggag tcgctgcgtt gccttcgccc cgtgccccgc tccgcgccgc

2641 ctcgcgccgc ccgccccggc tctgactgac cgcgttactc ccacaggtga gcgggcggga

2701 cggcccttct cctccgggct gtaattagcg cttggtttaa tgacggctcg tttcttttct

2761 gtggctgcgt gaaagcctta aagggctccg ggagggccct ttgtgcgggg gggagcggct

2821 cggggggtgc gtgcgtgtgt gtgtgcgtgg ggagcgccgc gtgcggcccg cgctgcccgg

2881 cggctgtgag cgctgcgggc gcggcgcggg gctttgtgcg ctccgcgtgt gcgcgagggg

2941 agcgcggccg ggggcggtgc cccgcggtgc gggggggctg cgaggggaac aaaggctgcg

3001 tgcggggtgt gtgcgtgggg gggtgagcag ggggtgtggg cgcggcggtc gggctgtaac

3061 ccccccctgc acccccctcc ccgagttgct gagcacggcc cggcttcggg tgcggggctc

3121 cgtgcggggc gtggcgcggg gctcgccgtg ccgggcgggg ggtggcggca ggtgggggtg

3181 ccgggcgggg cggggccgcc tcgggccggg gagggctcgg gggaggggcg cggcggcccc

3241 ggagcgccgg cggctgtcga ggcgcggcga gccgcagcca ttgcctttta tggtaatcgt

3301 gcgagagggc gcagggactt cctttgtccc aaatctggcg gagccgaaat ctgggaggcg

3361 ccgccgcacc ccctctagcg ggcgcgggcg aagcggtgcg gcgccggcag gaaggaaatg

3421 ggcggggagg gccttcgtgc gtcgccgcgc cgccgtcccc ttctccatct ccagcctcgg

3481 ggctgccgca gggggacggc tgccttcggg ggggacgggg cagggcgggg ttcggcttct

3541 ggcgtgtgac cggcggctct agagcctctg ctaaccatgt tcatgccttc ttctttttcc

3601 tacagatcct taattaaccg tcttaagcct gtaacaaccg gtacagttcg aataacttcg

3661 tatagcatac attatacgaa gttatgaagt tataccgggc caccatagtc gcgagtagct

3721 tgggccagct aggccttgac caaagttcct ctggaattgc gatcgcacgc gtatgacgta

3781 cttcccggag gaggtggtgg agcacatctt cagcttcctg ccggcgcagc gcgaccgcaa

3841 cacggtctcg ctcgtctgca aggtgtggta cgagatcgag aggctgagcc gccgcggcgt

3901 cttcgtgggc aactgctacg ccgtgcgcgc cggccgcgtc gccgcgcggt tccccaacgt

3961 gcgggcgctc acggtgaagg ggaagcccca cttcgccgac ttcaacctcg tgccccccga

4021 ctggggcggc tacgcggggc cgtggatcga ggcggccgcg aggggatgcc acggcctgga

4081 ggagctcagg atgaagcgga tggtggtgtc cgacgagagc ctcgagctgc tggctcgctc

4141 gttcccgcgg ttcagggctc ttgttcttat cagctgcgag gggttcagca ctgacgggct

4201 agccgccgtc gcgagccatt gcaagcttct gagggagttg gatttgcagg aaaatgaagt

4261 ggaggatcga gggcctaggt ggctttcctg cttccctgat tcctgcacat cacttgtctc

4321 attgaatttt gcctgcatca aaggggaggt taatgctggt tcactggaga gacttgttag

4381 caggtcccca aacctgcgga gtttgaggct gaatcgatct gtatcggtag atacacttgc

4441 aaagatacta ctgcgtaccc ctaacttgga ggatttgggg acagggaatt tgacagatga

4501 cttccaaact gagtcctact ttaagcttac cagtgctctg gagaaatgca agatgttgag

4561 gagtttgtct ggattctggg atgcttctcc tgtttgcctg tcatttatct accccctgtg

4621 tgctcaactg acaggattga acttgagcta tgcacccaca cttgatgctt ctgaccttac

4681 aaaaatgatt agccgctgtg tgaagctcca acgcctttgg gtactggatt gtatctcgga

4741 caaaggcttg caagtggtgg cctccagttg caaagacttg caagaactca gggtatttcc

4801 atcagatttc tacgtagctg gttattctgc agtgacagag gagggacttg ttgcagtatc

4861 cttgggctgt ccaaaactga actcactact gtacttctgt caccaaatga ctaatgctgc

4921 actagttact gtcgccaaga actgtccaaa tttcacacga ttcagacttt gtattcttga

4981 gccagggaag cctgatgttg tgacaagcca accattagat gaaggctttg gagctattgt

5041 tcgtgagtgc aagggattac aacgtttgtc aatatctggt cttctcacag acaaagtttt

5101 catgtatatt gggaaatatg caaaacaact tgagatgctt tctatagcat ttgctggtga

5161 cagtgataag ggtatgatgc atgttatgaa tggatgcaag aatttaagga aactggagat

5221 aagagatagc ccgtttggtg atgctgcact cttggggaat tttgctaggt acgagacaat

5281 gcgatccctt tggatgtcat cttgcaatgt cacgttaaag gggtgccaag tccttgcgtc

5341 aaagatgccg atgctcaatg ttgaggtcat aaatgagcgg gatggtagca atgaaatgga

5401 ggaaaaccat ggagatctgc ctaaagtgga gaaattatat gtgtaccgca caactgctgg

5461 ggcgagggat gatgcaccaa attttgttaa aatcctagtc gactccggtt ctgctgctag

5521 tggtgaacaa aagttgattt ctgaagaaga tttgaacggt gaacaaaagc taatctccga

5581 ggaagacttg aacggtgaac aaaaattaat ctcagaagaa gacttgaacg gatccactag

5641 ctaaacgcgt aaatgattgc agatccacta gttctagagc tcgctgatca gcctcgactg

5701 tgccttctag ttgccagcca tctgttgttt gcccctcccc cgtgccttcc ttgaccctgg

5761 aaggtgccac tcccactgtc ctttcctaat aaaatgagga aattgcatcg cattgtctga

5821 gtaggtgtca ttctattctg gggggtgggg tggggcagga cagcaagggg gaggattggg

5881 aagacaatag caggcatgct ggggatgcgg tgggctctat ggcttctgag gcggaaagaa

5941 ccagctgggg ctcgagcctc tagaactata gtgagtcgta ttacgtagat ccagacatga

6001 taagatacat tgatgagttt ggacaaacca caactagaat gcagtgaaaa aaatgcttta

6061 tttgtgaaat ttgtgatgct attgctttat ttgtaaccat tataagctgc aataaacaag

6121 ttaacaacaa caattgcatt cattttatgt ttcaggttca gggggaggtg tgggaggttt

6181 tttaattcgc ggccctagaa gatgggcggg agtcttctgg gcaggcttaa aggctaacct

6241 ggtgtgtggg cgttgtcctg caggggaatt gaacaggtgt aaaattggag ggacaagact

6301 tcccacagat tttcggtttt gtcgggaagt tttttaatag gggcaaataa ggaaaatggg

6361 aggataggta gtcatctggg gttttatgca gcaaaactac aggttattat tgcttgtgat

6421 ccgcctcgga gtattttcca tcgaggtaga ttaaagacat gctcacccga gttttatact

6481 ctcctgcttg agatccttac tacagtatga aattacagtg tcgcgagtta gactatgtaa

6541 gcagaatttt aatcattttt aaagagccca gtacttcata tccatttctc ccgctccttc

6601 tgcagcctta tcaaaaggta ttttagaaca ctcattttag ccccattttc atttattata

6661 ctggcttatc caacccctag acagagcatt ggcattttcc ctttcctgat cttagaagtc

6721 tgatgactca tgaaaccaga cagattagtt acatacacca caaatcgagg ctgtagctgg

6781 ggcctcaaca ctgcagttct tttataactc cttagtacac tttttgttga tcctttgcct

6841 tgatccttaa ttttcagtgt ctatcacctc tcccgtcagg tggtgttcca catttgggcc

6901 tattctcagt ccagggagtt ttacaacaat agatgtattg agaatccaac ctaaagctta

6961 actttccact cccatgaatg cctctctcct ttttctccat ttataaactg agctattaac

7021 cattaatggt ttccaggtgg atgtctcctc ccccaatatt acctgatgta tcttacatat

7081 tgccaggctg atattttaag acattaaaag gtatatttca ttattgagcc acatggtatt

7141 gattactgct tactaaaatt ttgtcattgt acacatctgt aaaaggtggt tccttttgga

7201 atgcaaagtt caggtgtttg ttgtctttcc tgacctaagg tcttgtgagc ttgtattttt

7261 tctatttaag cagtgctttc tcttggactg gcttgactca tggcattcta cacgttattg

7321 ctggtctaaa tgtgattttg ccaagcttct tcaggaccta taattttgct tgacttgtag

7381 ccaaacacaa gtaaaatgat taagcaacaa atgtatttgt gaagcttggt ttttaggttg

7441 ttgtgttgtg tgtgcttgtg ctctataata atactatcca ggggctggag aggtggctcg

7501 gagttcaaga gcacagactg ctcttccaga agtcctgagt tcaattccca gcaaccacat

7561 ggtggctcac aaccatctgt aatgggatct gatgccctct tctggtgtgt ctgaagacca

7621 caagtgtatt cacattaaat aaataaatcc tccttcttct tctttttttt ttttttaaag

7681 agaatactgt ctccagtaga atttactgaa gtaatgaaat actttgtgtt tgttccaata

7741 tggtagccaa taatcaaatt actctttaag cactggaaat gttaccaagg aactaatttt

7801 tatttgaagt gtaactgtgg acagaggagc cataactgca gacttgtggg atacagaaga

7861 ccaatgcaga ctttaatgtc ttttctctta cactaagcaa taaagaaata aaaattgaac

7921 ttctagtatc ctatttgttt aaactgctag ctttacttaa cttttgtgct tcatctatac

7981 aaagctgaaa gctaagtctg cagccattac taaacatgaa agcaagtaat gataattttg

8041 gatttcaaaa atgtagggcc agagtttagc cagccagtgg tggtgcttgc ctttatgcct

8101 ttaatcccag cactctggag gcagagacag gcagatctct gagtttgagc ccagcctggt

8161 ctacacatca agttctatct aggatagcca ggaatacaca cagaaaccct gttggggagg

8221 ggggctctga gatttcataa aattataatt gaagcattcc ctaatgagcc actatggatg

8281 tggctaaatc cgtctacctt tctgatgaga tttgggtatt attttttctg tctctgctgt

8341 tggttgggtc ttttgacact gtgggctttc tttaaagcct ccttcctgcc atgtggtctc

8401 ttgtttgcta ctaacttccc atggcttaaa tggcatggct ttttgccttc taagggcagc

8461 tgctgagatt tgcagcctga tttccagggt ggggttggga aatctttcaa acactaaaat

8521 tgtcctttaa tttttttttt aaaaaatggg ttatataata aacctcataa aatagttatg

8581 aggagtgagg tggactaata ttaaatgagt ccctccccta taaaagagct attaaggctt

8641 tttgtcttat acttaacttt ttttttaaat gtggtatctt tagaaccaag ggtcttagag

8701 ttttagtata cagaaactgt tgcatcgctt aatcagattt tctagtttca aatccagaga

8761 atccaaattc ttcacagcca aagtcaaatt aagaatttct gacttttaat gttaatttgc

8821 ttactgtgaa tataaaaatg atagcttttc ctgaggcagg gtctcactat gtatctctgc

8881 ctgatctgca acaagatatg tagactaaag ttctgcctgc ttttgtctcc tgaatactaa

8941 ggttaaaatg tagtaatact tttggaactt gcaggtcaga ttcttttata ggggacacac

9001 taagggagct tgggtgatag ttggtaaaat gtgtttcaag tgatgaaaac ttgaattatt

9061 atcaccgcaa cctacttttt aaaaaaaaaa gccaggcctg ttagagcatg cttaagggat

9121 ccctaggact tgctgagcac acaagagtag ttacttggca ggctcctggt gagagcatat

9181 ttcaaaaaac aaggcagaca accaagaaac tacagttaag gttacctgtc tttaaaccat

9241 ctgcatatac acagggatat taaaatattc caaataatat ttcattcaag ttttccccca

9301 tcaaattggg acatggattt ctccggtgaa taggcagagt tggaaactaa acaaatgttg

9361 gttttgtgat ttgtgaaatt gttttcaagt gatagttaaa gcccatgaga tacagaacaa

9421 agctgctatt tcgaggtctc ttggtttata ctcagaagca cttctttggg tttccctgca

9481 ctatcctgat catgtgctag gcctacctta ggctgattgt tgttcaaata aacttaagtt

9541 tcctgtcagg tgatgtcata tgatttcata tatcaaggca aaacatgtta tatatgttaa

9601 acatttgtac ttaatgtgaa agttaggtct ttgtgggttt gatttttaat tttcaaaacc

9661 tgagctaaat aagtcatttt tacatgtctt acatttggtg gaattgtata attgtggttt

9721 gcaggcaaga ctctctgacc tagtaaccct acctatagag cactttgctg ggtcacaagt

9781 ctaggagtca agcatttcac cttgaagttg agacgttttg ttagtgtata ctagtttata

9841 tgttggagga catgtttatc cagaagatat tcaggactat ttttgactgg gctaaggaat

9901 tgattctgat tagcactgtt agtgagcatt gagtggcctt taggcttgaa ttggagtcac

9961 ttgtatatct caaataatgc tggccttttt taaaaagccc ttgttcttta tcaccctgtt

10021 ttctacataa tttttgttca aagaaatact tgtttggatc tccttttgac aacaatagca

10081 tgttttcaag ccatattttt tttccttttt tttttttttt ttggtttttc gagacagggt

10141 ttctctgtat agccctggct gtcctggaac tcactttgta gaccaggctg gcctcgaact

10201 cagaaatccg cctgcctctg cctcctgagt gccgggatta aaggcgtgca ccaccacgcc

10261 tggctaagtt ggatattttg ttatataact ataaccaata ctaactccac tgggtggatt

10321 tttaattcag tcagtagtct taagtggtct ttattggccc ttcattaaaa tctactgttc

10381 actctaacag aggctgttgg tactagtggc acttaagcaa cttcctacgg atatactagc

10441 agattaaggg tcagggatag aaactagtct agcgttttgt atacctacca gctttatact

10501 accttgttct gatagaaata tttcaggaca tctagcttat cgataccgtc gacggtatcg

10561 ataagcttga tccagctttt gttcccttta gtgagggtta attgcgcgct tggcgtaatc

10621 atggtcatag ctgtttcctg tgtgaaattg ttatccgctc acaattccac acaacatacg

10681 agccggaagc ataaagtgta aagcctgggg tgcctaatga gtgagctaac tcacattaat

10741 tgcgttgcgc tcactgcccg ctttccagtc gggaaacctg tcgtgccagc tgcattaatg

10801 aatcggccaa cgcgcgggga gaggcggttt gcgtattggg cgctcttccg cttcctcgct

10861 cactgactcg ctgcgctcgg tcgttcggct gcggcgagcg gtatcagctc actcaaaggc

10921 ggtaatacgg ttatccacag aatcagggga taacgcagga aagaacatgt gagcaaaagg

10981 ccagcaaaag gccaggaacc gtaaaaaggc cgcgttgctg gcgtttttcc ataggctccg

11041 cccccctgac gagcatcaca aaaatcgacg ctcaagtcag aggtggcgaa acccgacagg

11101 actataaaga taccaggcgt ttccccctgg aagctccctc gtgcgctctc ctgttccgac

11161 cctgccgctt accggatacc tgtccgcctt tctcccttcg ggaagcgtgg cgctttctca

11221 tagctcacgc tgtaggtatc tcagttcggt gtaggtcgtt cgctccaagc tgggctgtgt

11281 gcacgaaccc cccgttcagc ccgaccgctg cgccttatcc ggtaactatc gtcttgagtc

11341 caacccggta agacacgact tatcgccact ggcagcagcc actggtaaca ggattagcag

11401 agcgaggtat gtaggcggtg ctacagagtt cttgaagtgg tggcctaact acggctacac

11461 tagaaggaca gtatttggta tctgcgctct gctgaagcca gttaccttcg gaaaaagagt

11521 tggtagctct tgatccggca aacaaaccac cgctggtagc ggtggttttt ttgtttgcaa

11581 gcagcagatt acgcgcagaa aaaaaggatc tcaagaagat cctttgatct tttctacggg

11641 gtctgacgct cagtggaacg aaaactcacg ttaagggatt ttggtcatga gattatcaaa

11701 aaggatcttc acctagatcc ttttaaatta aaaatgaagt tttaaatcaa tctaaagtat

11761 atatgagtaa acttggtctg acagttacca atgcttaatc agtgaggcac ctatctcagc

11821 gatctgtcta tttcgttcat ccatagttgc ctgactcccc gtcgtgtaga taactacgat

11881 acgggagggc ttaccatctg gccccagtgc tgcaatgata ccgcgagacc cacgctcacc

11941 ggctccagat ttatcagcaa taaaccagcc agccggaagg gccgagcgca gaagtggtcc

12001 tgcaacttta tccgcctcca tccagtctat taattgttgc cgggaagcta gagtaagtag

12061 ttcgccagtt aatagtttgc gcaacgttgt tgccattgct acaggcatcg tggtgtcacg

12121 ctcgtcgttt ggtatggctt cattcagctc cggttcccaa cgatcaaggc gagttacatg

12181 atcccccatg ttgtgcaaaa aagcggttag ctccttcggt cctccgatcg ttgtcagaag

12241 taagttggcc gcagtgttat cactcatggt tatggcagca ctgcataatt ctcttactgt

12301 catgccatcc gtaagatgct tttctgtgac tggtgagtac tcaaccaagt cattctgaga

12361 atagtgtatg cggcgaccga gttgctcttg cccggcgtca atacgggata ataccgcgcc

12421 acatagcaga actttaaaag tgctcatcat tggaaaacgt tcttcggggc gaaaactctc

12481 aaggatctta ccgctgttga gatccagttc gatgtaaccc actcgtgcac ccaactgatc

12541 ttcagcatct tttactttca ccagcgtttc tgggtgagca aaaacaggaa ggcaaaatgc

12601 cgcaaaaaag ggaataaggg cgacacggaa atgttgaata ctcatactct tcctttttca

12661 atattattga agcatttatc agggttattg tctcatgagc ggatacatat ttgaatgtat

12721 ttagaaaaat aaacaaatag gggttccgcg cacatttccc cgaaaagtgc cacctaaatt

12781 gtaagcgtta atattttgtt aaaattcgcg ttaaattttt gttaaatcag ctcatttttt

12841 aaccaatagg ccgaaatcgg caaaatccct tataaatcaa aagaatagac cgagataggg

12901 ttgagtgttg ttccagtttg gaacaagagt ccactattaa agaacgtgga ctccaacgtc

12961 aaagggcgaa aaaccgtcta tcagggcgat ggcccactac gtgaaccatc accctaatca

13021 agttttttgg ggtcgaggtg ccgtaaagca ctaaatcgga accctaaagg gagcccccga

13081 tttagagctt gacggggaaa gccggcgaac gtggcgagaa aggaagggaa gaaagcgaaa

13141 ggagcgggcg ctagggcgct ggcaagtgta gcggtcacgc tgcgcgtaac caccacaccc

13201 gccgcgctta atgcgccgct acagggcgcg tcccattcgc cattcaggct gcgcaactgt

13261 tgggaagggc gatcggtgcg ggcctcttcg ctattacgcc agctggcgaa agggggatgt

13321 gctgcaaggc gattaagttg ggtaacgcca gggttttccc agtcacgacg ttgtaaaacg

13381 acggccagtg agcgcgcgta atacgactca ctatagggcg aattggagct

//
